# Association of the resolvin precursor 17-HDHA, but not D- or E- series resolvins, with heat pain sensitivity and osteoarthritis pain in humans

**DOI:** 10.1038/s41598-017-09516-3

**Published:** 2017-09-07

**Authors:** Ana M. Valdes, Srinivasarao Ravipati, Cristina Menni, Abhishek Abhishek, Sarah Metrustry, Juliette Harris, Ayrun Nessa, Frances M. K. Williams, Tim D. Spector, Michael Doherty, Victoria Chapman, David A. Barrett

**Affiliations:** 1 Department of Twin Research and Genetic Epidemiology, King’s College London, London, UK; 2 Academic Rheumatology Clinical Sciences Building, Nottingham City Hospital, Hucknall Road, Nottingham, UK; 3 National Institute of Health Research, Nottingham Biomedical Research Centre, Nottingham, UK; 4 Centre for Analytical Bioscience, School of Pharmacy, University of Nottingham, NG7 2RD Nottingham, UK; 5 School of Life Sciences, University of Nottingham, Nottingham, UK; 6 Arthritis Research UK Centre of Excellence for Pain, University of Nottingham, Nottingham, UK

## Abstract

Resolvins are omega-3 fatty acid derived potent bioactive lipids that resolve inflammation and modulate transient receptor potential channels. Exogenous administration of the resolvin precursor 17-HDHA shows a strong analgesic effect in animal models of osteoarthritis and acute inflammatory pain, but has not been studied in humans. Our aim was to assess the role of 17-HDHA and resolvins in heat pain sensitivity and in osteoarthritis pain in humans. Resolvins D1, D2, D3, D5, E1 and 17-HDHA, were measured by liquid chromatography-mass spectrometry and tested for association with heat pain thresholds in 250 healthy volunteers who had undergone quantitative sensory testing. Resolvins D1, D2 and 17-HDHA were then tested in 62 individuals affected with knee osteoarthritis and 52 age matched controls and tested for association with knee pain. Circulating levels of docosahexaenoic acid (DHA) were also measured. Levels of 17-HDHA, but not those of the other 5 resolvins tested, were associated with increased heat pain thresholds (beta = 0.075; 95% CI 0.024, 0.126; p < 0.0046). 17-HDHA was associated with lower pain scores in OA patients (beta −0.41; 95% CI-0.69, −0.12; p < 0.005; adjusted for covariates) but not with radiographic osteoarthritis. The associations of 17-HDHA with heat pain sensitivity and osteoarthritis pain were independent of DHA levels.

## Introduction

Osteoarthritis (OA) is the fastest growing cause of chronic pain worldwide and is the commonest form of arthritis^1^. Pain is the predominant symptom of OA, which limits movement and causes disability^2^. It has long been recognised that the amount of pain people with OA experience is variable and only weakly correlated to the extent of joint damage, but the molecular basis underlying variation in OA pain is poorly understood^2^. It is well established pro-inflammatory agents are released into the joint and that synovitis is highly correlated to OA pain^3^, various cytokines and pro-inflammatory mediators are released in damaged tissue and induce a cascade of events that lead to peripheral sensitization^4^. These mediators accumulate in the joint as OA progresses^4^ and act to reduce threshold of joint nociceptors.

**Figure 1:**
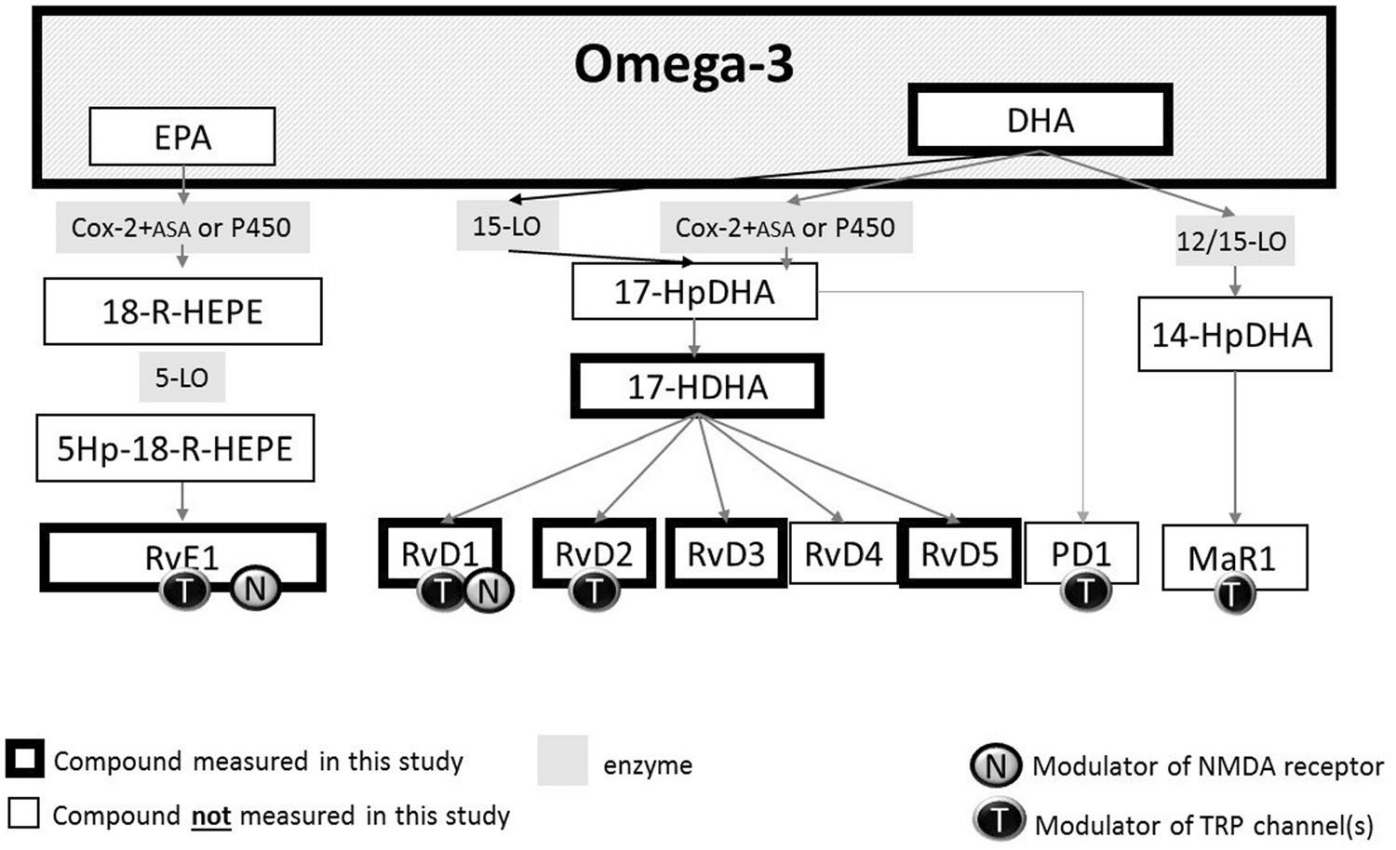
Relationship between the omega-3 lipids measured in this study and other omega 3-fatty acid derived oxylipins. The assays did not distinguish chirality therefore compounds measure can be either the 17-S or 17-R enantiomers (e.g. 17-S-HDHA or 17-R-HDHA). Abbreviations: HEPE = Hydroxyeicosapentaenoic acid; RvE1 = resovlin E1; RvD(x) = resolvin Dx; PD1 = protectin D1; MaR1 = maresin 1; EPA = Eicosapentaenoic acid;DHA Docosahexaenoic acid; COX-2 + ASA = cycloxygenase -2 and aspirin; HpDHA = hydroperoxy docosahexaenoic acid; HDHA = hydroxy docosahexaenoic acid; OxoDHA = oxodocosahexaenoic acid. TRP = transient potential receptor; NMDA = N-Methyl-D-Aspartic acid.

Chronic localised inflammatory pain is a feature of several chronic diseases^5^. The role of specialised pro-resolvin molecules in curtailing inflammatory responses has been demonstrated in cell systems and *in vivo*, with a fundamental role in the maintenance of tissue homeostasis^6^. The best characterised of these resolution molecules are the resolvins, which are oxygenated metabolites (or oxylipins) of omega-3 fatty acids^7^. The omega-3 fatty acids docosahexaenoic acid (DHA) and eicosapentaenoic acid (EPA) are the precursors of D and E-series resolvins, respectively^6^ (Fig. 1). Resolvins suppress cytokine production and “resolve” the destructive inflammation^7^ by regulating leukocyte trafficking and clearance of inflammatory mediators (e.g. cytokines, prostaglandins and leukotrienes, growth factors and reactive oxygen species). D- and E-series resolvins inhibit several transient receptor potential (TRP) channels *in vitro* ^5, 8^ which are essential to cutaneous thermal and pain sensation^9, 10^. Thus, in addition to their effect on inflammatory mediators, these compounds may play a direct role in nociception and specifically on thermal pain. Resolvins are also able to modulate N-Methyl-D-Aspartic acid (NMDA) receptors^11^ (Fig. 1). Phosphorylation of these receptors has been associated with hyperalgesia, neuropathic pain, and reduced functionality of opioid receptors^12^.

Exogenous administration of resolvin E1 (RvE1) and resolvin D1 (RvD1) inhibits pain behaviour in animal models of acute inflammatory pain^8^ and in a model of chronic adjuvant-induced arthritis^13^.

RvD1 reduces mechanical allodynia and phosphorylation of NMDA receptors along with decreased cytokine expression in an animal model of chronic pancreatitis^11^. Moreover, RvD1 has been shown to significantly attenuate arthritis severity and inflammation in a murine model of inflammatory arthritis but these protective actions are abolished in RvD1 receptor-deficient mice^14^.

Resolvins are rapidly degraded *in vivo* resulting in a short biological half-life, but this limitation can be overcome by administration of precursors of the active molecules^13^. 17-HDHA is a metabolite of the omega-3 fatty acid docosahexaenoic acid (DHA), and a precursor of D-series resolvins. Inhibitory effects of exogenous omega-3 fatty acids on pain and inflammation is evident in rheumatoid arthritis^15^, however a randomised clinical trial of high vs low dose omega-3 fatty acids failed to show an advantage of high omega-3 administration^16^.

Exogenous 17-HDHA halted progression of pain behaviour in a surgical model of OA pain^17^. However, 17-HDHA did not alter the extent of joint pathology, pointing to potential direct effects on sensory nerves or in the central nervous system and suggesting the therapeutic potential of this new class of analgesics for the treatment of OA pain. Thus, measurement of 17-HDHA may be useful in pinpointing the potential mechanisms of action of omega-3 on pain, which may reflect the effect of various D-series resolvins.

The potential role of resolvins and their metabolic precursors in human pain has yet to be explored, partly due to the lack of analytical methods for analysis of these lipids in biological tissues. Recent availability of authentic chemical standards for the resolvins and precursors has allowed the development of highly sensitive and specific liquid chromatography tandem mass spectrometry methods (LC-MS/MS) for their quantitative analysis^18^. The aim of our study was to assess the role of 17-HDHA and resolvins in pain sensitivity, both in acute heat pain and in OA pain in humans. To address this aim, we have firstly investigated if the D-series resolvin precursor 17-HDHA is associated with thermal pain sensitivity *per se* in human volunteers, and whether any association with heat pain sensitivity can be explained by the levels of D- or E- series resolvins. Whether possible associations between 17-HDHA and pain sensitivity were reflective of omega-3 dietary intake was also established. Given the known role of inflammatory mediators in OA pain^4^ and potential link of pain responses to inflammation we also included in our model two omega-6 lipids, the prostaglandin precursor arachidonic acid (AA) and the leukotriene B4 recently reported to be involved in peripheral sensitization^19^. We then asked if 17-HDHA or D-series resolvins were associated with knee pain intensity in individuals with knee OA, and whether any putative associations with OA pain are reflective of DHA serum levels. Finally, we assessed whether levels of 17-HDHA were associated with OA status comparing individuals affected with radiographic knee OA to OA free controls.

## Subjects and Methods

Details on the study cohorts are included in the Supplementary section. All methods in this study were performed in accordance with relevant guidelines and regulations. Briefly:

### Subjects

#### TwinsUK cohort

The TwinsUK registry contains twin volunteers recruited through national media campaigns and from other twin registers. The study was approved by the St Thomas’ Hospital research ethics committee, and all participants provided written informed consent. Participants be comparable to the age-matched general population singletons for a broad variety of medical and behavioural traits^20^. Unselected twins (n = 2500) had been invited to attend St Thomas’ Hospital where they completed questionnaires gathering demographic information, clinical history and current medications. Exclusions included volunteers who had consumed analgesic medication within 12 hours of the study visit, and those with likely impaired upper limb neurology, e.g. known neuropathy, previous stroke or chemotherapy. Subjects having common painful conditions such as OA were not excluded^21^. Participants underwent sensory testing individually as previously described^21^. The heat pain threshold (HPT) represents the temperature at which the sensation evoked by a thermal stimulus changes from feeling ‘hot’ to feeling ‘painful’ while the heat pain super threshold (HPST) records the temperature at which the sensation changes from “painful to unbearable”. Venous blood was taken after the pain threshold testing (see below) and serum was extracted and stored at −80C. A subset of 250 samples was transferred to the School of Pharmacy in Nottingham. Five resolvins, one resolvin precursor, plus arachidonic acid and leukotriene B4 were measured in these samples. (see below).

#### Osteoarthritis case-control cohort

62 individuals affected with radiographic knee OA (defined as Kellgren Lawrence grade of 2 or higher)^22^ and 52 individuals with no radiographic nor clinical symptoms of OA were recruited from existing databases of previous OA studies at the University of Nottingham. Approval for recruitment was obtained from the research ethics committees of Nottingham City Hospital and North Nottinghamshire. Bilateral knee radiographs of index knee or hip OA cases were obtained at two time points and scored for features of OA by a single observer using the Kellgren and Lawrence grade for the tibio-femoral and patella femoral compartments of each knee^22^. Individuals also donated a blood sample from which plasma was extracted and stored for further analysis, and underwent a pain assessment. Participants completed a detailed nurse administered questionnaire on their symptoms and quality of life. Pain assessment was performed with the Western Ontario and McMaster Universities Arthritis Index (WOMAC)^23^ was used to quantify knee pain. Plasma samples were transferred for lipidomic analyses were carried that determine plasma concentration levels of the resolvin precursor (17-HDHA) and resolvins D1 and D2, plus arachidonic acid (AA) and leukotriene B4 (LTB4). In addition, levels of DHA and total omega-3 in the plasma samples were measured using the methods described below.

### Laboratory analyses

#### Oxylipin and resolvin analysis method

The LC-MS/MS method used for eicosanoid analysis in human serum samples was based on the method previously developed^24, 25^ adapted for the inclusion of resolvins and precursors as described in the Supplementary Section. All lipid measurements were quantitative and had precision values of better than ±15% and accuracies in the range 85–115% as determined from the analysis of calibration and QCs with each batch of samples.

*Quantification* was performed using fully extracted calibration standards for each of the analytes. Measured concentrations of 17-HDHA, Resolvin D2, Resolvin D1, Resolvin D3, Resolvin D5, Resolvin E1, AA, LTB4 were detectable in each sample and are corrected for sample volume where appropriately needed.

#### NMR Metabolomics

DHA and total omega 3 fatty acids were measured by Brainshake Ltd, Finland, (https://www.brainshake.fi/) from fasting serum or plasma samples using 500 MHz and 600 MhH proton nuclear magnetic resonance spectroscopy as previously described^26^.

### Statistical analysis

Concentrations of all resolvins, DHA and omega-3 fatty acids were log-transformed for analysis. The association between bioactive lipids and heat pain thresholds in TwinsUK was carried out using linear regressions using the log transformed HPT and HSPT as the outcome and adjusting for family relatedness, age and BMI. Additional models including circulating levels of DHA were also tested. Association between log transformed WOMAC pain scores and plasma resolvins and DHA were adjusted for age, sex, BMI and K/L grade or OA status. Analyses were performed using R version 3.0.1 (www.cran.org).

## Results

The descriptive characteristics of the participants of both cohorts are summarized in Table 1. Plasma levels of 17-HDHA and D-series resolvins (OA case control) were lower than serum levels (TwinsUK cohort). The relationship between the omega-3 compounds measured is shown in Fig. 1.

**Table 1:** Descriptive characteristics of study cohorts.

cohort	TwinsUK		Nottingham controls		Nottingham knee OA	
n	250		52		62	
sex F	100%		57.9%		61.1%	
age mean (SD)	67.42	(8.08)	67.78	(7.35)	67.96	(8.62)
BMI kg/m^2^ mean (SD)	26.55	(4.40)	28.24	(5.12)	31.11	(6.43)
HPT °C mean (SD)	46.26	(2.24)				
HPST °C mean (SD)	47.46	(1.84)				
K/L grade 0|1|2|3|4	NA		0.81|0.19| 0|0|0		0 | 0 | 0.11|0.42|0.47	
WOMAC (0–20) pain score mean (SD)	NA		2.17	(3.70)	6.93	(4.37)
***Serum bioactive lipids mean (SD)***						
RvD1(pmol/mL)	0.042	(0.089)	*NA*		*NA*	
RvD2 (pmol/mL)	2.78	(3.34)	*NA*		*NA*	
17-HDHA (pmol/mL)	162.32	(277.47)	*NA*		*NA*	
RvD3 (pmol/mL)	0.23	(0.53)	*NA*		*NA*	
RvD5 (pmol/mL)	0.39	(1.39)	*NA*		*NA*	
RvE1 (pmol/mL)	0.68	(2.32)	*NA*		*NA*	
DHA (mmol/L)	0.14	(0.05)	*NA*		*NA*	
total omega 3 (mmol/L)	0.43	(0.13)	*NA*		*NA*	
AA (pmol/L)	5531.10	(1949.4)	*NA*		*NA*	
LTB4 (pmol/mL)	79.44	(212.64)	*NA*		*NA*	
***Plasma bioactive lipids mean (SD)***						
RvD1(pmol/mL)	*NA*		0.022	(0.026)	0.22	(0.029)
RvD2 (pmol/mL)	*NA*		1.24	(1.90	0.74	(0.86)
17-HDHA (pmol/mL)	*NA*		26.98	(18.56	25.40	(19.06)
DHA (mmol/L)	*NA*		0.13	(0.02)	0.13	(0.02)
total omega 3 (mmol/L)	*NA*		0.34	(0.08)	0.33	(0.07)
AA (pmol/L)			3012.6	(1553.6)	3133.38	(1335.5)
LTB4 (pmol/mL)			14.86	(24.87)	10.27	(10.93)

BMI = body mass index; HPT = heat pain threshold; HPST = heat pain super thresold; WOMAC = Western Ontario & McMaster Universities Osteoarthritis Index; K/L grade = Kellgren- Lawrence radiographic grade. RvDx = resolvin Dx; DHA = docohexadecanoic acid; AA = arachidonic acid. LTB4 = leukotriene B4.

### Association with heat pain thresholds

We first tested if there was an association between the omega-3 derived bioactive lipids and heat pain sensitivity after adjustment for potential confounders. Of the lipids quantified, only 17-HDHA was associated with both HPST and HPT. None of the other D-series resolvins, or resolvin E1, were associated with pain sensitivity in this cohort. DHA and total omega −3 levels were associated with HPST but not HPT (Table 2). Adjustment for circulating levels of DHA did not alter the association between 17-HDHA and heat pain thresholds (HPT and HPST) (Table 2).

**Table 2:** Association between serum resolvins and omega-3 fatty acids and heat pain thresholds (HPT) and heat pain super threshold (HPST) in the TwinsUK cohort. Association is expressed as the regression coefficient (beta) and the corresponding 95% confidence intervals (95% CI). The outcomes tested were HPT and HPST in the log scale, resolvin measures were also log transformed and regressons are adjusted for age, BMI and relatedness.

	logHPT				log HPST			
	beta	95% CI		p-value	beta	95% CI		p-value
RvD1	−0.005	(−0.046, 0.036)		0.805	−0.046	(−0.120, 0.028)		0.231
RvD2	0.021	(−0.006, 0.048)		0.133	0.000	(−0.051, 0.051)		0.988
**17-HDHA**	**0.035**	**(0.008, 0.062)**		**0.012**	**0.075**	**(0.024, 0.126)**		**0.0046**
RvD3	−0.023	(−0.050, 0.004)		0.100	−0.032	(−0.083, 0.019)		0.231
RvD5	−0.017	(−0.046, 0.012)		0.242	−0.033	(−0.086, 0.020)		0.226
RvE1	0.003	(−0.026, 0.032)		0.834	−0.017	(−0.072, 0.038)		0.545
DHA	0.017	(−0.016, 0.050)		0.329	**0.030**	**(0.003, 0.057)**		**0.031**
Total omega3	0.017	(−0.016, 0.050)		0.323	**0.028**	**(0.003, 0.053)**		**0.036**
AA	0.0001	(−0.002, 0.003)		0.921	0.002	(−0.004, 0.007)		0.571
LTB4	0.001	(−0.003, 0.005)		0.558	−0.001	(−0.007, 0.005)		0.752
17-HDHA adj^(1)^	**0.033**	**0.006**	**0.060**	**0.018**	**0.071**	**0.016**	**0.126**	**0.011**
DHA adj^(1)^	0.014	−0.019	0.047	0.410	**0.029**	**0.002**	**0.056**	**0.038**

^1^Model includes both DHA and 17-HDHA in addition to age, BMI and family relatedness. Nominally significant associations are highlighted in bold.

Overall, DHA and omega-3 fatty acids were poorly associated with the various resolvins and this only achieved statistical significance for 17-HDHA and RvD2 (Supplementary Table 1). The pro-inflammatory mediator AA was negatively correlated with RvD2, RvD5, RvE1 and LTB4. LTB4 on the other hand was positively correlated with all the resolvins measured, except RvD2. However, we found no association between AA and LTB4 levels and heat pain thresholds. (Table 2).

We also assessed the correlations between the various resolvin compounds with each other, and with age and BMI (Supplementary Table 1). Although some resolvins were strongly correlated with each other, 17-HDHA was only significantly (and modestly) correlated with RvD2. The resolvins had no association with BMI, and only a weak correlation with age (Supplementary Table 1).

**Figure 2:**
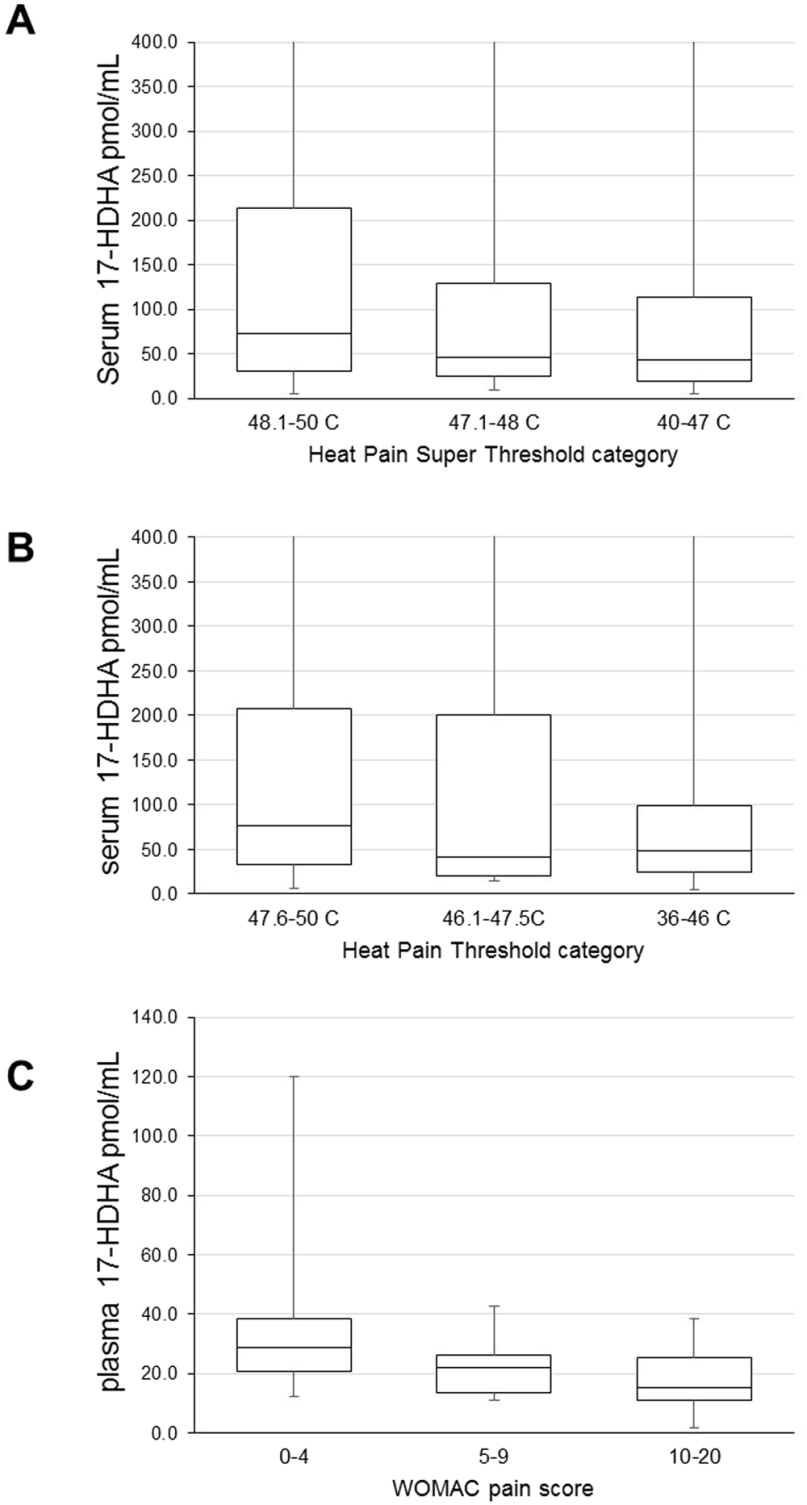
Distribution of serum (**A** and **B**) and plasma (**C**) 17-HDHA by categories of pain sensitivity and pain severity. HPT = heat pain threshold HPST = heat pain super threshold, WOMAC = Western Ontario and McMaster Universities Arthritis Index (0 = no knee pain, 20 = highest level of knee pain).

The distribution of the serum 17-HDHA levels in TwinsUK divided by categories of heat pain thresholds and heat pain super thresholds in the natural scale are shown in Fig. 2(A and B). Adjusting for the levels of LTB4 and AA had no effect on the association between 17-HDHA and HPT and HPST. The association between 17-HDHA and logHPT after adjusting for LTB4 and AA was beta = 0.0035 (95% CI 0.0008–0.0063 P = 0.0124), for logHPST it was beta = 0.0032 (95%CI 0.0010–0.0055; P = 0.005).

### Association of 17-HDHA with OA and OA pain

Since 17-HDHA was associated with pain sensitivity in humans, we hypothesized that this compound and not other D-series resolvins would be associated with OA pain. Herein we demonstrate that 17-HDHA, but not resolvin D1 nor D2, was associated with pain scores overall (Table 3). This was the case both in cases and controls adjusting for OA status and in OA cases only (unadjusted for radiographic severity first and then adjusted for radiographic severity) (Table 3). Moreover, we found no association between levels of any of the three resolvins and OA case control status (Supplementary Table 2). The distribution of plasma levels of 17-DHDA in the natural scale in three WOMAC pain scores categories in OA patients and is shown graphically in Fig. 2C.

**Table 3:** Association between plasma resolvins, omega-3 fatty acids and OA pain in the Nottingham cohort. The outcome of all regressions is the log-transformed WOMAC pain score (+1). A higher WOMAC score indicates more main. Regressions were first run only in OA patients adjusting for age, sex, BMI and K/L grade. In selected models both 17-HDHA and DHA or total omega 3 were tested for association together, finally association between 17-HDHA and WOMAC scores was tested in the combined case-control group adjusting for OA status in addition to all other covariates.

compound (log)	beta	95% CI		p-value
RvD1	0.022	−0.033	0.078	0.430
RvD2	0.029	−0.095	0.152	0.649
**17-HDHA**	−**0.406**	−**0.690**	−**0.123**	**0.005**
total omega3	−0.375	−0.775	0.024	0.065
**DHA**	−**1.316**	−**2.532**	−**0.100**	**0.034**
(DHA + 17-HDHA in same model)				
**17-HDHA**	−**0.396**	−**0.686**	−**0.105**	**0.008**
DHA	−1.282	−2.582	0.018	0.053
(omega 3 + 17-HDHA in same model)				
**17-HDHA**	−**0.401**	−**0.682**	−**0.121**	**0.005**
Total omega3	−0.352	−0.779	0.074	0.106
**17-HDHA adj for LTB4**	−**0.364**	−**0.678**	−**0.051**	**0.024**
**17-HDHA adj for AA**	−**0.460**	−**0.779**	−**0.141**	**0.005**
(OA + controls)				
**log17-HDHA**	−**0.123**	−**0.234**	−**0.012**	**0.029**

Taken together the heat pain sensitivity data and the OA pain data suggest that circulating 17-HDHA is a compound directly involved in pain. However, omega-3 fatty acids are known to have anti-inflammatory and anti-nociceptive effects and therefore this association of 17-HDHA may simply reflect the effect of omega-3 fatty acids. To explore whether this is the case, we tested the association of 17-HDHA with pain scores adjusting for levels of DHA and for omega-3. DHA was significantly associated with pain scores independently of 17-HDHA, but the association between 17-HDHA remains significant and unaltered when including DHA or omega-3 in the model and the DHA effect is close to nominal significance even including 17-HDHA (Table 3). These data suggest that there may be two separate effects on pain and that the effect of 17-HDHA is not the one commonly attributed to omega-3/DHA. (Table 3) Finally, we adjusted results for the circulating levels of two omega-6 derived lipids (AA and LTB4) and the association between 17-HDHA and OA pain remained similar (Table 3).

## Discussion

In this study we show for the first time that 17-HDHA is associated with pain in humans, specifically with thermal pain sensitivity and the intensity of chronic pain. Previously, we have shown that exogenous systemic administration of 17-hydroxy docosahexaenoic acid (17-HDHA also abbreviated as 17-HDoHE) completely reversed established pain behaviour in the monosodium iodoacetate model of OA pain, an effect which lasted for 6 hours, and was sustained following repeated administration^17^.

We report that the relationship with two measures of heat pain threshold in the general population and with pain severity in OA patients is specific for the resolvin precursor 17-HDHA, and not evident for other D-series resolvins. The association between 17-HDHA and the three pain measures tested was not attenuated by adjustment for DHA, total omega-3, arachidonic acid or leukotriene B4 levels. An association between DHA and HPST and OA pain was seen, but not with HPT which remained statistically significant only in the case of HPST after adjustment for 17-HDHA. These results indicate that the relationship between 17-HDHA and pain levels is not reflective of levels of DHA, since both compounds have independent effects on pain. This is suggestive of two separate mechanisms are involved, consistent with *in vitro* reports of the various DHA derived oxylipins involved in pain In particular a DHA effect not related to levels of 17-HDHA could be explained by the action of maresin 1 (MaR1) which was not measured in our study (see Fig. 1).

The correlation between levels of DHA and 17-HDHA seen was only modest, which suggests that the antinociceptive effects of 17-HDHA are unlikely to be strongly modulated by omega-3 dietary supplementation, and may depend on the efficiency of the metabolic pathways (15-lipoxygenase for the 17-S isomers or COX-2 plus aspirin for the 17-R oxylipins) that convert DHA to 17-HDHA, and not to other metabolites None of the compounds tested were associated with OA case-control status consistent with the observation that repeated treatment with exogenous 17-HDHA does not alter joint pathology in a rat model of OA^17^. This is different from the situation seen in animal models of rheumatoid arthritis, where D-series resolvins are strongly associated with joint pathology^14^.

Further studies are needed to understand the mechanisms by which 17-HDHA mediates an analgesic effect. One possibility is that higher levels of 17-HDHA lead to higher levels of 17-HpDHA, which in turn can be epoxidized into protectin D1 (Fig. 1). Although this mechanism could explain the human data it is harder to reconcile with the strong and long lasting analgesia produced by exogenous administration of 17-HDHA in rodent models of OA^17^. Another possibility is that the inhibitory effects of 17-HDHA are all due to RvD4 (not measured in our study but known to modulate receptors involved in nociception), or that 17-HDHA may be directly involved in modulating pain and pain sensitivity.

Transient receptor potential (TRP) family of ligand-gated ion channels, in particular TRPV1, have an established role in thermal sensitivity^9^ and contributes to pain in OA (e.g. ref. 27). Previous studies have reported effects of the resolvins on TRP channels. In an *in vitro* dorsal root ganglia (sensory nerve) preparation RvD1 suppressed the activity of various TRP channels (TRPA1, TRPV3 and TRPV4), but not TRPV1 and TRPV2 activity. TRPV1 activity was however inhibited by RvE1^5^. D- and E-series resolvins also modulate NMDA receptors in animal models^12^ and reduce mechanical allodynia and pain behaviour in models of chronic pancreatitis^11^. However, in our human study neither RvD1 nor RvE1 are associated with thermal pain thresholds. To our knowledge the role of 17-HDHA on TRP channels has not been reported in the literature^12^, therefore it is feasible that 17-HDHA may have direct effects on TRP function. Additional mechanisms by which 17-HDHA may exert an effect on pain includes a metabolite of 17-HDHA, 17-oxoDHA which is an agonist at peroxisome proliferator activated receptors (PPAR) alpha and gamma^28^. Interestingly, PPAR-alpha and gamma agonists have anti-nociceptive effects in a rat model of irritable bowel syndrome via a nitric-oxide mediated mechanism^29^.

Our study highlights for the first time the potential of 17-HDHA as a biomarker for pain and provides a platform for a potential new approach to analgesia. Nevertheless, to separate 17-S and 17-R isomers of the various compounds would enable further mechanistic insight. For example, levels of RvD2 could be reflecting more activity via the 17-S (15 lipoxygenase mediated) pathway whereas levels of 17-HDHA may be reflecting the 17-R (P450 or COX-2+ aspirin or statin mediated) pathway. We did not test other DHA derived bioactive lipids such as maresins and protectins^6^ which are likely to have an effect on pain responses (Fig. 1). Although we did not measure EPA levels, we did measure an EPA derived resolvin (RvE1) and found no association with heat pain thresholds.

One of the benefits of our study is the demonstration that 17-HDHA can be measured both in plasma and serum samples in humans. However, we do report large differences between the mean values of 17-HDHA and the two D-series resolvins measured in plasma (OA case control study) and serum samples. Although serum and heparinized plasma specimens are considered equivalent for some assays, differences in results between sample types have been reported for many chemistry analytes^30^. The higher levels in serum are consistent with what is known about resolvin and resolvin precursors (i.e. the presence of platelets generates higher concentration of these lipids^31^. It is important to note that the twin cohort in which we have tested the relationship between resolvins were only females. However, the heat pain thresholds data have been used successfully in other studies and have been shown to be heritable^21^. Finally, the OA cohort was only assessed for the standard OA pain instrument (WOMAC) and not for pain thresholds.

In conclusion, we have shown that 17-HDHA is associated with pain in humans, specifically with heat pain sensitivity and the intensity of chronic pain, which is unlikely to be due to its being a precursor to other D-series resolvins. 17-HDHA is known to be anti-nociceptive in models of OA pain and our novel finding that 17-HDHA can modulate pain responses in humans suggest that exogenous administration of 17-HDHA in humans may also be analgesic. Further work is needed to understand the anti-nociceptive mechanism of 17-HDHA which will shed light on pathways suitable for therapeutic intervention in OA pain.

## Electronic supplementary material

Supplementary Info

## References

[1] Glyn-Jones S (2015). Osteoarthritis. Lancet.

[2] Perrot S (2015). Osteoarthritis pain. Best Pract Res Clin Rheumatol.

[3] Berenbaum F (2013). Osteoarthritis as an inflammatory disease (osteoarthritis is not osteoarthrosis!). Osteoarthritis Cartilage.

[4] Krustev E, Rioux D, McDougall JJ (2015). Mechanisms and Mediators That Drive Arthritis Pain. Curr Osteoporos Rep.

[5] Ji RR, Xu ZZ, Strichartz G, Serhan CN (2011). Emerging roles of resolvins in the resolution of inflammation and pain. Trends Neurosci.

[6] Serhan CN (2014). Pro-resolving lipid mediators are leads for resolution physiology. Nature.

[7] Schwanke RC, Marcon R, Bento AF, Calixto JB (2016). EPA- and DHA-derived resolvins’ actions in inflammatory bowel disease. Eur J Pharmacol.

[8] Bang S, Yoo S, Yang TJ, Cho H, Hwang SW (2012). 17(R)-resolvin D1 specifically inhibits transient receptor potential ion channel vanilloid 3 leading to peripheral antinociception. Br J Pharmacol.

[9] Mickle, A. D., Shepherd, A. J. & Mohapatra, D. P. Nociceptive TRP Channels: Sensory Detectors and Transducers in Multiple Pain Pathologies. *Pharmaceuticals (Basel)* **9** (2016). 10.3390/ph9040072 PMC5198047 27854251

[10] Cortright DN, Szallasi A (2009). TRP channels and pain. Curr Pharm Des.

[11] Quan-Xin F (2012). Resolvin D1 reverses chronic pancreatitis-induced mechanical allodynia, phosphorylation of NMDA receptors, and cytokines expression in the thoracic spinal dorsal horn. BMC Gastroenterol.

[12] Choi G, Hwang SW (2016). Modulation of the Activities of Neuronal Ion Channels by Fatty Acid-Derived Pro-Resolvents. Front Physiol.

[13] Yoo S, Lim JY, Hwang SW (2013). Resolvins: Endogenously-Generated Potent Painkilling Substances and their Therapeutic Perspectives. Curr Neuropharmacol.

[14] Norling LV (2016). Proresolving and cartilage-protective actions of resolvin D1 in inflammatory arthritis. JCI Insight.

[15] Lee YH, Bae SC, Song GG (2012). Omega-3 polyunsaturated fatty acids and the treatment of rheumatoid arthritis: a meta-analysis. Arch Med Res.

[16] Hill CL (2016). Fish oil in knee osteoarthritis: a randomised clinical trial of low dose versus high dose. Ann Rheum Dis.

[17] Huang J (2017). Targeting the D Series Resolvin Receptor System for the Treatment of Osteoarthritis Pain. Arthritis Rheumatol.

[18] Colas RA, Shinohara M, Dalli J, Chiang N, Serhan CN (2014). Identification and signature profiles for pro-resolving and inflammatory lipid mediators in human tissue. Am J Physiol Cell Physiol.

[19] Zinn S (2017). The leukotriene B4 receptors BLT1 and BLT2 form an antagonistic sensitizing system in peripheral sensory neurons. J Biol Chem.

[20] Moayyeri A, Hammond CJ, Valdes AM, Spector TD (2013). Cohort Profile: TwinsUK and healthy ageing twin study. Int J Epidemiol.

[21] Williams FM (2012). Genes contributing to pain sensitivity in the normal population: an exome sequencing study. PLoS Genet.

[22] Kellgren JH, Lawrence JS, Bier F (1963). Genetic Factors in Generalized Osteo-Arthrosis. Ann Rheum Dis.

[23] Bellamy N, Buchanan WW (1984). Outcome measurement in osteoarthritis clinical trials: the case for standardisation. Clin Rheumatol.

[24] Zhang JH (2007). Quantitative profiling of epoxyeicosatrienoic, hydroxyeicosatetraenoic, and dihydroxyeicosatetraenoic acids in human intrauterine tissues using liquid chromatography/electrospray ionization tandem mass spectrometry. Anal Biochem.

[25] Wong A (2014). Simultaneous tissue profiling of eicosanoid and endocannabinoid lipid families in a rat model of osteoarthritis. J Lipid Res.

[26] Soininen P, Kangas AJ, Wurtz P, Suna T, Ala-Korpela M (2015). Quantitative serum nuclear magnetic resonance metabolomics in cardiovascular epidemiology and genetics. Circ Cardiovasc Genet.

[27] Valdes AM (2011). The Ile585Val TRPV1 variant is involved in risk of painful knee osteoarthritis. Ann Rheum Dis.

[28] Egawa D, Itoh T, Akiyama Y, Saito T, Yamamoto K (2016). 17-OxoDHA Is a PPARalpha/gamma Dual Covalent Modifier and Agonist. ACS Chem Biol.

[29] Paragomi P (2014). Antinociceptive and antidiarrheal effects of pioglitazone in a rat model of diarrhoea-predominant irritable bowel syndrome: role of nitric oxide. Clin Exp Pharmacol Physiol.

[30] Miles RR, Roberts RF, Putnam AR, Roberts WL (2004). Comparison of serum and heparinized plasma samples for measurement of chemistry analytes. Clin Chem.

[31] Lannan KL, Spinelli SL, Blumberg N, Phipps RP (2017). Maresin 1 induces a novel pro-resolving phenotype in human platelets. J Thromb Haemost.

