# Supplementary material for: Association of the resolvin precursor 17-HDHA, but not D- or E- series resolvins, with heat pain sensitivity and osteoarthritis pain in humans
Source: Sci Rep. 2017 Sep 7;7:10748. doi: 10.1038/s41598-017-09516-3 (PMC5589894; doi:10.1038/s41598-017-09516-3)
Supplement: Supplementary file 1 — Supplementary Info [file 41598_2017_9516_MOESM1_ESM.doc]

**Association of the resolvin precursor 17-HDHA, but not D- or E- series resolvins, with heat pain sensitivity and osteoarthritis pain in humans.**

Ana M Valdes, Srinivasarao Ravipati, Cristina Menni, Abhishek Abhishek, Sarah Metrustry, Juliette Harris,, Ayrun Nessa, Frances MK Williams, Tim D Spector, Michael Doherty, Victoria Chapman, David A. Barrett

## SUPPLEMENTARY SECTION

Contents

[Study Subjects 2](#__RefHeading___Toc476297449)

[Oxylipin and resolvin analysis method: 3](#__RefHeading___Toc476297450)

[Supplementary Table 1. 6](#__RefHeading___Toc476297451)

[Supplementary Table 2. 6](#__RefHeading___Toc476297452)

## Study Subjects

*TwinsUK cohort*

The TwinsUK registry contains twin volunteers recruited through national media campaigns and from other twin registers. They are not selected for any particular trait. Subjects on the register take part in studies that cover a wide range of traits and common medical conditions and in general are not aware of the precise hypothesis being tested. The study was approved by the St Thomas’ Hospital research ethics committee, and all participants provided written informed consent. Twins from this registry have been shown to be comparable to the age-matched general population singletons for a broad variety of medical and behavioural traits ^1^. Unselected twins (n=2500) had been invited to attend St Thomas’ Hospital where they completed questionnaires gathering demographic information, clinical history and current medications. Exclusions included volunteers who had consumed analgesic medication within 12 hours of the study visit, and those with likely impaired upper limb neurology, e.g. known neuropathy, previous stroke or chemotherapy. Subjects having common painful conditions such as OA were not excluded^2^. Venous blood was taken after the pain threshold testing (see below) and serum was extracted and stored at -80C. A subset of 250 samples were transferred to the School of Pharmacy in Nottingham. Five resolvins and one resolvin precursor were measured in these samples.

*Quantitative Sensory testing (QST)*

Participants underwent sensory testing individually as previously described ^2^. A 25mm^2^ x 50mm^2^ probe connected to a Modular Sensory Analyzer Thermal Stimulator (Somedic, Sweden) was secured with a fabric-covered band on the volar surface of the forearm. Subjects received standardized instructions before both assessments. The heat pain threshold (HPT) represents the temperature at which the sensation evoked by a thermal stimulus changes from feeling ‘hot’ to feeling ‘painful’ while the heat pain super threshold (HPST) records the temperature at which the sensation changes from “painful to unbearable”. HPT was measured by heating the probe (rate of 0.5^0^C/s) from an adaptation temperature of 32^0^C until the subject perceived the stimulus as changing from hot to painful and stopped the experiment by pressing a button, at which point the temperature (equivalent to HPT) was automatically logged and the probe temperature returned to 32^0^C. If the subject reached 50^0^C the machine automatically returned to adaptation temperature to prevent thermal burn. A ‘practice run’ at measuring HPT was followed (10 seconds later), by the true HPT measurement. HPST was determined after the probe had been removed to the opposite arm. The probe was heated from 32^0^C (1^0^C/s) until the subject perceived the stimulus as changing from “painful to unbearable” and stopped the experiment, logging the temperature (equivalent to HPST). If the probe reached 50^0^C the machine automatically returned to adaptation temperature to prevent thermal burn. HPST was assessed without a trial run.

*Osteoarthritis case-control cohort*

62 individuals with radiographic OA and 52 free from radiographic OA were recruited from existing databases of previous OA studies (which had included X-ray assessments) at the University of Nottingham. Approval for recruitment was obtained from the research ethics committees of Nottingham City Hospital and North Nottinghamshire. Subjects were excluded from the study if they had other major arthropathy (e.g. rheumatoid arthritis, ankylosing spondylitis); Paget’s disease of bone affecting the pelvis or femur; overt childhood hip disease (e.g. Legg-Calve-Perthe’s disease, slipped femoral epiphysis, severe acetabular dysplasia); or if they had undergone already a total knee replacement. Height and weight were measured to calculate BMI. Controls were age and sex matched individuals from the same hospital catchment area. Bilateral knee radiographs of index knee or hip OA cases were obtained at two time points and scored for features of OA by a single observer using the Kellgren and Lawrence grade for the tibio-femoral and patella femoral compartments of each knee^3^ . Individuals also donated a blood sample from which plasma was extracted and stored for further analysis, and underwent a pain assessment. Participants completed a detailed nurse administered questionnaire on their symptoms and quality of life. Pain assessment was performed with the Western Ontario and McMaster Universities Arthritis Index (WOMAC)^4^ was used to quantify knee pain. Plasma samples were transferred to the School of Pharmacy (D. Barrett) where lipidomic analyses were carried that determine plasma concentration levels of the resolvin precursor (17-HDHA ) resolvins D1 and D2, arachidonic acid and leukotriene B4. In addition, levels of DHA and total omega-3 in the plasma samples were measured using the methods described below.

## Oxylipin and resolvin analysis method:

All methods in this study were performed in accordance with relevant guidelines and regulations.

The LC-MS/MS method used for eicosanoid analysis in human serum samples was based on the method previously developed ^5,6^adapted for the inclusion of resolvins and precursors.

*Equipment***:** The HPLC system used was a Shimadzu series 10AD VP LC system (Shimadzu, Columbia, MD, USA). The HPLC Column used was ACE C18 (150 x 2.1 mm I.D, 3 µm particle size) with guard column (Security Guard Cartridges ACE 3 C18 for ID 150 x 2.1 mm column). Mobile phase A was 0.02% formic acid in methanol:acetonitrile (1:4, v/v); mobile phase B was 0.02% formic acid in 100% water. The starting flow rate was 200 µl/min. Strata-X polymeric SPE column (200mg/6 ml) were purchased from Phenomenex, Macclesfield, UK. The evaporator used was a Jouan centrifugal evaporator (Saint-Herblain, France). The MS system used was an Applied Biosystem MDS SCIEX 4000 Q-Trap hybrid triple-quadrupole–linear ion trap mass spectrometer (Applied Biosystem, Foster City, CA, USA) equipped with an electrospray ionisation (ESI) interface.

*Standards***:** Resolvin D1, Resolvin D2, (±)17-hydroxy-4Z, 7Z, 10Z, 13Z, 15E, 19Z-docosahexaenoic acid (17-HDHA), Resolvin D3, Resolvin D5, Resolvin E1, 10S,17S-diHDHA, Resolvin D2- d5, arachidonic acid and leukotriene B4 were purchased from Cayman Chemicals (MI, USA).

One batch of blank human plasma (for the OA case-control) or serum (for the TwinsUK study) acted as an analytical quality control was used to confirm the day-to-day accuracy/precision of the method during the analysis of each batch of sample analysis.

*Extraction Protocol for Samples:* Samples were stored at -80°C before analysis. An internal standard of 10 µl of Resolvin D2-d5 (4 µM) was added to each sample or blank sample (0.4 ml water), along with 2 µl of formic acid (98% v/v) and 5 µl of an antioxidant butylhydroxytoluene (BHT). Samples were homogenised in micro centrifuge tubes with the addition of 900 µl of ethanol, followed by a slow vortex stage (10 min) and centrifuged (13000 g, 10 min, 4 °C). The supernatants were transferred to glass tubes and diluted by the addition of 3ml water. The diluted supernatants were loaded to the Strata-X polymeric SPE column (200mg/6 ml, Phenomenex, Macclesfield, UK) that had been preconditioned with 100% ethanol (2ml) and 25% ethanol (4 ml). The SPE cartridge then washed with distilled water (10 ml) and 25% ethanol (5 ml) and was allowed to run it dry. Then the eicosanoids were eluted from the column with ethyl acetate containing 0.0002%BHT (5 ml) and were dried in centrifugal evaporator. The samples were reconstituted in 50% ethanol (100 µl) and transferred to an auto sampler vial prior to LC-MS/MS analysis. The injection volume was 20 µl.

*Quantification* was performed using fully extracted calibration standards for each of the analytes. Quantification was performed using Analyst 1.4.1. Identification of each compound in serum samples was confirmed by LC retention times of each standard and precursor and product ion *m/z* ratios. The peak area of each analyte was compared with a known amount of standard to determine the amount of target compound present. Traits were log-transformed for statistical analysis

Measured concentrations of 17-HDHA, Resolvin D2, Resolvin D1, Resolvin D3, Resolvin D5, Resolvin E1, arachidonic acid, and leukotriene B4 were detectable in each sample and are corrected for sample volume where appropriately needed.

*NMR Metabolomics:* DHA and total omega 3 fatty acids were measured by Brainshake Ltd, Finland, (https://www.brainshake.fi/) from fasting serum or plasma samples using 500 MHz and 600 MhH proton nuclear magnetic resonance spectroscopy as previously described^7^ .

.

## Supplementary Table 1.

Correlation between resolvins, total omega 3 (n-3) fatty acids, DHA, arachidonic acid (AA), leukotriene B4 (LTB4), age and BMI. The Pearson’s correlation coefficient and p-values are shown.

| **Trait** | correlation | **RvD1** | **RvD2** | **17-HDHA** | **RvD3** | **RvD5** | **RvE1** | **AA** | **LTB4** |
| --- | --- | --- | --- | --- | --- | --- | --- | --- | --- |
| **RvD2** | rho | -0.167 |  |  |  |  |  |  |  |
|  | P | 0.092 |  |  |  |  |  |  |  |
| **17-HDHA** | rho | 0.01 | **0.296** |  |  |  |  |  |  |
|  | P | 0.92 | **1.7E-05** |  |  |  |  |  |  |
| **RvD3** | rho | **0.548** | 0.045 | 0.068 |  |  |  |  |  |
|  | P | **5.7E-11** | 0.532 | 0.34 |  |  |  |  |  |
| **RvD5** | rho | **0.579** | 0.178 | -0.088 | **0.541** |  |  |  |  |
|  | P | **1.30E-12** | 0.012 | 0.22 | **2.80E-19** |  |  |  |  |
| **RvE1** | rho | **0.453** | 0.061 | 0.048 | **0.603** | **0.62** |  |  |  |
|  | P | **3.6E-06** | 0.421 | 0.523 | **8.1E-24** | **1.0E-25** |  |  |  |
| **AA** | rho | -0.038 | **-0.191** | 0.055 | -0.024 | **-0.309** | **-0.218** |  |  |
|  | P | 0.651 | **0.0024** | 0.382 | 0.699 | **3.0E-07** | **0.0011** |  |  |
| **LTB4** | rho | **0.272** | 0.053 | **0.158** | **0.356** | **0.134** | **0.31** | **-0.222** |  |
|  | P | **0.001** | 0.48 | **0.012** | **2.7E-09** | **0.035** | **1.0E-06** | **4.1E-04** |  |
| **omega-3** | rho | 0.067 | **0.188** | **0.158** | 0.013 | 0.028 | -0.065 | 0.123 | 0.005 |
|  | P | 0.513 | **0.009** | **0.014** | 0.86 | 0.696 | 0.391 | 0.069 | 0.947 |
| **DHA** | rho | 0.117 | 0.107 | **0.144** | 0.01 | 0.065 | -0.049 | 0.127 | -0.041 |
|  | P | 0.25 | 0.139 | **0.046** | 0.886 | 0.371 | 0.517 | 0.063 | 0.554 |
| **age** | rho | **0.227** | 0.072 | **0.175** | **0.156** | **0.16** | 0.014 | 0.085 | -0.011 |
|  | p | **0.017** | 0.325 | **0.014** | **0.028** | **0.025** | 0.857 | 0.176 | 0.867 |
| **BMI** | rho | -0.095 | 0.024 | 0.028 | -0.077 | -0.081 | -0.013 | 0.005 | 0.106 |
|  | p | 0.18 | 0.736 | 0.695 | 0.279 | 0.257 | 0.868 | 0.935 | 0.094 |

## Supplementary Table 2.

Lack of associaton between 17-HDHA, resolvins D1 and D2, and DHA levels with OA . Association between DHA and derived metabolites and OA was computed by logistic regression adjusted for age, sex and BMI

| **compound** | **odds ratio** | **95% CI** |  | **p-value** |
| --- | --- | --- | --- | --- |
| 17-HDHA | 1.09 | (0.56, | 2.14) | 0.80 |
| RvD1 | 0.96 | (0.75, | 1.24) | 0.77 |
| RvD2 | 0.44 | (0.17, | 1.14) | 0.09 |
| DHA | 0.89 | (0.06, | 14.01) | 0.94 |

1. Moayyeri, A., Hammond, C.J., Valdes, A.M. & Spector, T.D. Cohort Profile: TwinsUK and healthy ageing twin study. *Int J Epidemiol* **42**, 76-85 (2013).

2. Williams, F.M.*, et al.* Genes contributing to pain sensitivity in the normal population: an exome sequencing study. *PLoS Genet* **8**, e1003095 (2012).

3. Kellgren, J.H., Lawrence, J.S. & Bier, F. Genetic Factors in Generalized Osteo-Arthrosis. *Ann Rheum Dis* **22**, 237-255 (1963).

4. Bellamy, N. & Buchanan, W.W. Outcome measurement in osteoarthritis clinical trials: the case for standardisation. *Clin Rheumatol* **3**, 293-303 (1984).

5. Zhang, J.H.*, et al.* Quantitative profiling of epoxyeicosatrienoic, hydroxyeicosatetraenoic, and dihydroxyeicosatetraenoic acids in human intrauterine tissues using liquid chromatography/electrospray ionization tandem mass spectrometry. *Anal Biochem* **365**, 40-51 (2007).

6. Wong, A.*, et al.* Simultaneous tissue profiling of eicosanoid and endocannabinoid lipid families in a rat model of osteoarthritis. *J Lipid Res* **55**, 1902-1913 (2014).

7. Soininen, P., Kangas, A.J., Wurtz, P., Suna, T. & Ala-Korpela, M. Quantitative serum nuclear magnetic resonance metabolomics in cardiovascular epidemiology and genetics. *Circ Cardiovasc Genet* **8**, 192-206 (2015).
